# Modification of Cellulose Acetate Microfiltration Membranes Using Graphene Oxide–Polyethyleneimine for Enhanced Dye Rejection

**DOI:** 10.3390/membranes13020143

**Published:** 2023-01-22

**Authors:** Maria Dominique Ong, Isabel Vasquez, Brandon Alvarez, Dylan R. Cho, Malik B. Williams, Donovan Vincent, Md. Arafat Ali, Nirupam Aich, Alexandre H. Pinto, Mahbuboor Rahman Choudhury

**Affiliations:** 1 Civil and Environmental Engineering Department, Manhattan College, Riverdale, NY 10471, USA; 2 Chemistry and Biochemistry Department, Manhattan College, Riverdale, NY 10471, USA; 3 Department of Civil, Structural and Environmental Engineering, University at Buffalo, SUNY, Buffalo, NY 14228, USA; 4 Nebraska Center for Materials & Nanoscience, University of Nebraska—Lincoln, Lincoln, NE 68588, USA

**Keywords:** microfiltration membranes, graphene oxide, cellulose acetate, polyethhyleneimine, methylene blue, eriochrome black T

## Abstract

Pressure-based membrane processes represent excellent water resource recovery prospects from industrial waste streams. In contrast with conventional pretreatment technologies, studies have shown that membrane pretreatment applications, such as microfiltration (MF), are more cost-effective and improve the results of the overall treatment processes. Hence, enhancing rejection efficiency of MF will enhance the performance of any downstream treatment processes. In this study, 0.45 µm cellulose acetate (CA) microfiltration membranes were modified by vacuum filtration-assisted layer-by-layer deposition of bilayers composed of negatively charged graphene oxide (GO) and positively charged polyethyleneimine (PEI). The performance of 1-, 2-, and 4-bilayer GO–PEI-modified membranes were investigated for their dye-rejection of anionic eriochrome black T (EBT) dye and cationic methylene blue (MB) dye in a cross-flow membrane module. As the number of bilayers on the membrane increased, the membrane thicknesses increased, and the deionized (DI) water flux through the membranes decreased from 4877 LMH/bar for the control (no bilayer) membrane to 2890 LMH/bar for the 4-bilayer membrane. Conversely, the dye-rejection performance of the modified membranes increased as increasing bilayers of GO–PEI deposited on the membranes. The anionic EBT dye saw superior rejection (~90% rejection) compared to the cationic MB dye (~80% rejection), which can be attributable to the electrostatic repulsion between the negatively charged GO surface and anionic EBT dye. After 50% recovery of the saline and dye-laden feed water, there was an observed drop in DI water fluxes of ~40–41% and 36%, respectively. There was also a slight increase in EBT dye-rejection during the composite feed-water experiments, attributed to the precipitation of salts on the membrane feed side or pore spaces, which subsequently reduce the membrane pore sizes.

## 1. Introduction

While the textile industry is known to positively affect global economic development, it is notorious for its excessive water consumption [1]. Reports indicate that the textile industry is responsible for approximately 20 percent of global industrial wastewater, with the water demand for 1 kg of product requiring about 200 L of water [2,3]. Apart from this excessive water consumption, the textile industry produces wastewater effluent that contains carcinogenic dyes and a very high salt content, usually containing sodium chloride (NaCl) or sodium sulfate (Na_2_SO_4_) [4].

Almost all dyes and chemicals are applied to textile substrates in aqueous forms in most of the fabric preparation steps (e.g., desizing, scouring, bleaching, mercerizing, etc.) [5]. Hence, over 100,000 synthetic dyes and over 700,000 tons of “dyestuff” are produced annually [6]. Meanwhile, an estimated 20 percent of industry-produced dyes are ultimately discharged into water resources without proper treatment [7]. This is of particular concern because dye-laden effluent wastewater is known to be highly toxic and has been shown to impart physiological changes to aquatic life, cause eutrophication, and contribute to unacceptable aesthetics [8]. The dyes in the textile industrial effluent alone are challenging to treat, due to their stability, resistance to biodegradation, and chemical structure, which allows them to resist degradation even with exposure to light, water, and chemicals [9,10]. Among an array of different textile dyes, in this research we studied a cationic dye, methylene blue (MB), and an anionic dye, eriochrome black T (EBT). Besides their overall charge and structural features, these two dyes were chosen due to their recognized toxicity. For instance, MB was reported to cause nausea, vomiting, and increasing heart rate upon acute exposure [11,12]. Whereas, EBT was reported to be able to penetrate across organ barriers and cause organ toxicity and cause lysis of red blood cells based on in vitro studies [13].

The quality and/or quantity of water resources are further aggravated by the effect of increasing extremes of climate, e.g., regional or localized droughts, and are more profound in areas with growing populations [14]. Hence, the environmental pollution from the textile industry constitutes a global threat to public health, establishing the way for new environmental restoration initiatives [9].

Recovery of wastewater for reuse within the textile industries can offset the demand from natural water resources. Conventionally, textile industrial effluent has been treated by traditional biological or chemical/physical methods, e.g., adsorption and coagulation [15]. However, these methods have recognized disadvantages in their high operating costs, sludge-handling problems, and, more importantly, their inefficiency in completely degrading dyes and complex organic compounds, which remain in the treated effluent [16]. Biological processes, such as aerated lagoons and activated sludge and trickling filters, on the other hand, are limiting due to their requirements of large process reactors, long treatment times, and microorganism sensitivities [17].

Membrane-based separation processes, which employ a membrane to separate contaminants from a feed solution based on opening sizes in the membrane, have shown great promise for water recovery from industrial waste streams [18]. These processes are in-line, do not require any start-up time, and are modular in nature, making them easy to scale up or down. The membrane-based recovery processes can be used in conjunction with one or more conventional treatment processes to reduce the large volume of effluent coming from the textile industries, which would otherwise contaminate nearby water resources. These membranes can be classified as microfiltration (MF), ultrafiltration (UF), nanofiltration (NF), and reverse osmosis (RO), having opening sizes of 0.1–10 µm, 1–100 nm, 0.1–10 nm, and less than 0.1 nm, respectively [18]. While RO is a mature process, known for its production of high-quality permeate from a feed stream, other membrane-based processes (MF, UF) are employed in series as a pretreatment to RO feed water [19,20,21]. In contrast with conventional pretreatment technologies, studies have shown that membrane pretreatment applications are more cost-effective and improve the overall results of the process [20]. MF and UF applied in tandem with RO has been recognized as one of the best combinations for water resource recovery [21]. Therefore, enhancing rejection efficiency of MF will enhance downstream RO performance and operating time.

Graphene derivatives are promising materials for membrane modification, due to their chemical stability and unique two-dimensional structures [22]. In particular, graphene oxide (GO) is able to be implemented layer-by-layer, providing it with the ability to form two-dimensional nanochannels between GO sheets that can act as pores for highly selective molecular and ionic transport of GO-based membranes used in filtration [22]. The nearly frictionless nature of the surface of the GO allows water molecules to flow quickly through the channels, allowing GO-based membranes to achieve a high water flux [23]. On a membrane surface, GO can also be implemented with different organic molecules, using the oxygen functional groups on the edge and basal planes of the GO to improve membrane selectivity, antibacterial properties, and resistance to fouling [19]. Introducing interactive forces, e.g., covalent bonds, between the GO sheets can reportedly improve the physical stability of GO-based membranes [22]. GO nanosheets, which are negatively charged, can be electrostatically bonded to the positively charged polyelectrolyte, polyethyleneimine (PEI) [7]. The PEI induces an increased surface charge on the membrane and is believed to improve salt rejections [23]. Homem et al. (2019) modified the surface of a polyethersulfone MF membrane by incorporating GO and PEI, and reported enhanced dye removal performance in a dead-end filtration module [7]. In a dead-end filtration module, the feeding flux is perpendicular to the permeation, causing fouling to accumulate at the membrane surface and thus resulting in lower filtration efficacy [24]. Therefore, there is a need to analyze the performance of such modified membranes in a cross-flow membrane module, which benefits from the tangential shear and subsequently reduces foulant deposition [25]. Dead-end filtration is applied to recuperate the deposits (also called “cake”) on the membrane surface. The cross-flow filtration, on the other hand, is applied when we want to prevent formation of the deposit on the membrane, and water recovery remains the main objective [26]. For large-scale water recovery application using pressure-based membranes, cross-flow filtration has reportedly performed better than the dead-end filtration process [27].

In this study, surfaces of cellulose acetate (CA) MF membranes (0.45 µm) were modified using different amounts of alternating GO suspension and PEI solution by a simple vacuum-filtration-assisted technique. The vacuum-filtration-assisted deposition method was adopted because of its easy application and the simple instrumentation requirement for the modification of the CA membranes, thus allowing facile fabrication of modified MF membranes for enhanced dye rejection. The rejection performance of feed water containing different concentrations of dyes and salts were evaluated in a cross-flow membrane module to reflect the salt and dye rejection performance achieved by the modified membranes. A systematic set of membrane performance tests were carried out to evaluate: (a) effect of GO–PEI content on the rejection of dyes by modified membranes, (b) change in GO–PEI-modified membrane permeability as a result of 50% recovery of feed solution containing salt or dye, and (c) effect of feed-water salinity on the dye rejection performance of the modified membranes.

## 2. Materials and Methods

### 2.1. Chemicals and Materials

Polyethyleneimine (PEI, average molecular weight (MW) of 70,000, 30% wt. aqueous solution) were acquired from BeanTown Chemical (Hudson, NH, USA). Sodium sulfate (Na_2_SO_4_) and sodium chloride (NaCl) salts were procured from VWR International and Aldon Corp SE (Avon, NY, USA), respectively. Graphite powder was purchased from Fisher Chemicals. Cellulose acetate microfiltration membranes (0.45 µm) were supplied by Advantec MFC, Inc. (Atlanta, GA, USA). Methylene blue (MB) and eriochrome black T (EBT) dyes were purchased from Sigma-Aldrich and J.T. Baker Chemical (VWR), respectively. Membrane cleaning solutions were prepared using sodium dodecyl sulfate (SDS), which was acquired from VWR International. The graphene oxide (GO) used in the membrane modification process was produced from graphite based on a modified Hummer’s method, as presented by Huang et al. [28] In summary, 0.5000 g of graphite powder were dissolved in a mixture of 45 mL sulfuric acid (H_2_SO_4_) and 5 mL phosphoric acid (H_3_PO_4_). Then, 2.5000 g of potassium permanganate (KMnO_4_) was added to this mixture. The resulting mixture was left stirring at room temperature for 72 h. After that time, the graphite oxidation by the KMnO_4_ was quenched by adding 50 mL of H_2_O_2_—3% solution and 20 mL of deionized water. This quenching process led to gas evolution from the reaction container, and at the end of the quenching process the reaction content was transferred to centrifuge tubes, centrifuged at 3500× *g* rpm for 3 min, the supernatant was discarded, and the precipitate was washed with hydrochloric acid (HCl) 1 mol·L^−1^, and centrifuged again at 3500× *g* rpm for 3 min. This process was repeated three times. After that, the washing followed by the centrifuging process was repeated with deionized water, for seven times. After the three washing cycles with HCl and seven washing cycles with deionized water, the supernatant had a pH of around 4. The precipitate was transferred to Petri dishes and left to dry at room temperature in front of the air supplied by a fan.

### 2.2. Membrane Modification

**Figure 1:**
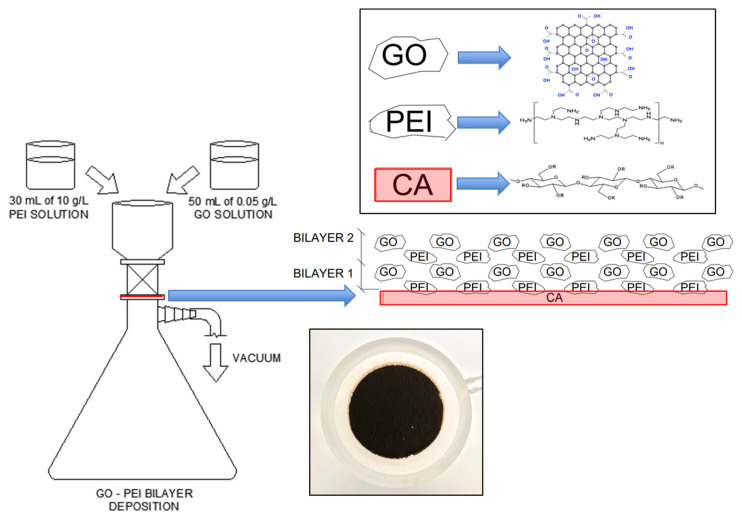
Cellulose acetate membrane modification process using graphene oxide–polyethyleneimine for dye/salt mixture solution.

Cellulose acetate (CA) membranes sized at 0.45 µm were modified with GO and PEI using a vacuum filtration setup. The membranes were modified by filtering 30 mL of PEI solution (10 g/L) through the CA membrane, and then filtering a 50 mL GO suspension (0.05 g/L) through the PEI-soaked CA membrane. The PEI-soaked membranes provide positively charged amine (-NH_2_) functional groups on the membrane surface. Subsequent filtering of the GO suspension on the membrane surface leads to electrostatic interaction between the positively charged amine groups on the PEI soaked membranes and negatively charged oxygen functional groups (e.g., carboxylic, alcohol, epoxide) in the GO particles (Figure 1).

This electrostatic interaction coupled with pressure deposition due to vacuum filtration produces one GO-PEI bilayer on the CA membrane. Similar alternating PEI solution and GO suspension solution were deposited using vacuum filtration to achieve 2- and 4-bilayers of GO-PEI on the CA membrane. A control CA membrane without any surface modification was also used to evaluate the dye rejection performance of the base membrane.

### 2.3. Membrane Characterization

#### 2.3.1. Fourier Transform Infrared Spectroscopy, X-ray Diffraction, Field Emission Scanning Electron Microscopy Analysis, and Zeta Potential Measurement

Fourier transform infrared spectroscopy (FTIR) of membranes was performed in attenuated total reflectance (ATR) mode, with 4 cm^−1^ resolution and 256 scans using a Thermo Scientific Nicollet 6700 FTIR spectrometer. The GO was characterized by powder X-ray diffraction using a Bruker D2 Phaser Powder X-ray Diffractometer, equipped with a CuKα radiation source. The XRD patterns were collected in a two-theta range from 2 to 120°, with an increment of 0.150° and 6 s per step. The total collection time was about 80 min. The microstructure of the GO particles was characterized using field emission scanning electron microscopy (FESEM, Zeiss Sigma VP SEM).

A 0.5 mg graphene oxide precursor (GO) sample was placed in a 50 mL aqueous solution and then it was ultrasonically dispersed in a bath-type sonicator for 20 min. The zeta potential of the GO sample was then measured using a Malvern Zetasizer Nano-ZS particle analyzer. At first, no pH adjustment was performed while measuring the zeta potential. Afterward, the suspension pH was adjusted within the range 2.5–11.55, using 0.1 M NaOH or 0.1 M HCl. For each sample, triplicate data were collected to obtain the average zeta potential with standard deviation.

#### 2.3.2. Surface and Thickness Analysis, Water Contact Angle Measurement

The membrane morphologies were observed using field emission scanning electron microscopy (FESEM, Zeiss Sigma VP SEM). The membrane thicknesses were measured using a digital Vernier caliper (electronic digital Vernier caliper, LOUISWARE, measurement accuracy 0.01 mm), where the modified membranes were placed between two microscope glass slides and the thickness of the glass slides, measured separately, were deducted from the Vernier caliper readings to obtain the thickness of the modified membranes. Static water contact angles were measured to estimate the hydrophilicity of the base membrane and surface-modified membranes. Twenty microliters of deionized (DI) water was dispensed on the membrane surface, and a picture taken from the edge of the membrane to measure the contact angle formed by the droplet on the membrane surface.

#### 2.3.3. Membrane Selectivity, Physical Stress Tests

To determine the membrane selectivity (molecular weight cutoff, MWCO), rejection tests were performed using a polyethylene glycol (PEG, MW = 20 KD, Alfa Aesar, Thermo Fisher Scientific, Heysham, UK) solution. A 1 g/L PEG solution was prepared and filtered through the modified membranes via vacuum filtration after precompaction with DI water for 30 min. The feed and permeate solutions were analyzed for chemical oxygen demand (COD) using Hach COD kit vials (CO, USA). Rejection was then calculated with the concentration of TOC present in permeate and feed solution.

The stability of the deposited GO–PEI layers was assessed by exposing the 2-bilayer-modified membrane to harsh physical stress [29]. The physical stress test involved immersing the modified membrane in 10 mL deionized water and bath sonicating it for 2 min. The membrane deposit was visually observed and the contact angle was measured after the physical stress test.

### 2.4. Membrane Experiments

**Figure 2:**
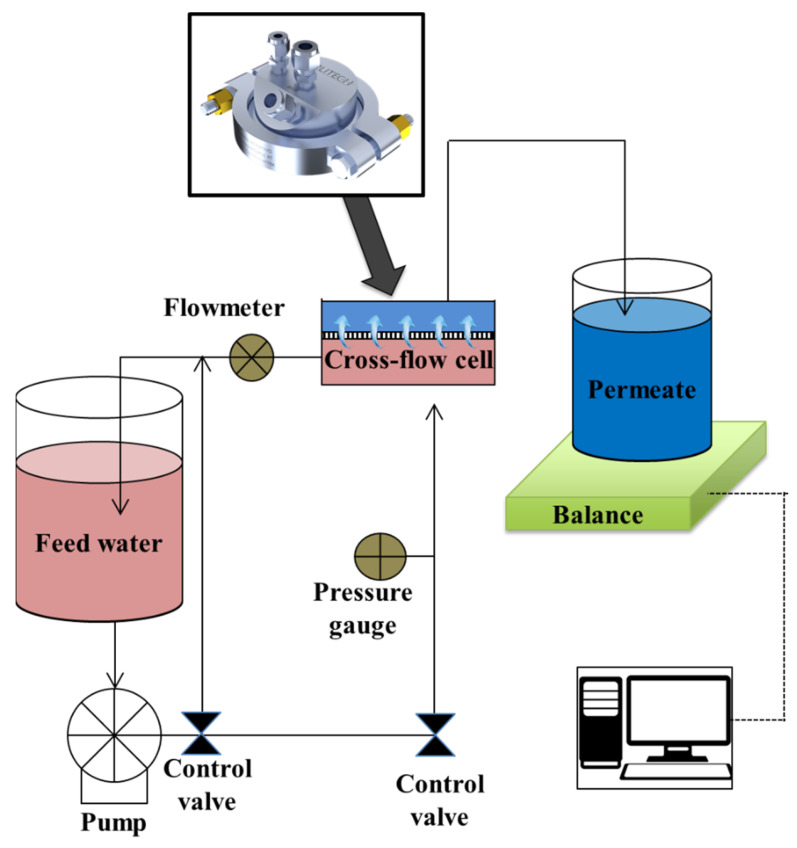
Schematic diagram of the cross-flow membrane module used in the membrane performance experiments.

The performance of the control and modified membranes in rejecting salts and dyes were evaluated in cross-flow mode using a stainless-steel cross-flow membrane cell (CF047 Circular Cell Assembly, Sterlitech, Auburn, WA, USA), which provides an effective membrane surface area of 14.6 cm^2^ on the feed side. Figure 2 shows the schematic layout of the cross-flow membrane experiment setup.

Prior to the rejection experiments, the membranes were precompressed using DI water for 60 min, which resulted in a steady flow of permeate through the membranes. Following the 60 min precompression period, the membranes were used with single-solute or multisolute feeds for dye and salt rejection. The feed-side pressure was maintained at about 14 psi and the cross-flow rate on the feed side was maintained at 0.35 gpm. The permeate flow rate was measured using a digital weighing scale (Quintix5102-1S, Sartorius, Bohemia, NY, USA), which recorded the weights at a 10 s interval. Solution permeability (J, LMH/bar) was calculated using the following equation:

$$J=\frac{\Delta V}{A\times \Delta t\times \Delta P}$$

where ΔV is the volume of permeate sample (L), A is the effective surface area of the membrane exposed to the feed water (m^2^), Δt is the duration of filtration (h), and ΔP is the transmembrane pressure (bar).

#### 2.4.1. Single-Solute Rejection Experiments

Single-solute rejection experiments involved using a dye or salt on the feed solution. This study used MB and EBT dyes in the feed solution at a concentration of 0.1 g/L. MB and EBT dyes have positive charge and negative charge, respectively. In addition, cross-flow salinity rejection experiments were operated using sodium sulfate or sodium chloride salt at a feed-side concentration of 1 g/L. The membrane rejection (R) was measured using the following equation:

$$R=\frac{C_{f}-C_{p}}{C_{f}}\times 100\%$$

where $C_{f}$ and $C_{p}$ are the feed and permeate solute concentrations, respectively. The salt concentrations were measured by an electrical conductivity meter (Oakton 6+ Series Conductivity Meter, Cole-Parmer, US), and the dye concentrations were measured with a UV-Vis spectrophotometer (Agilent 8453) by following the intensity of the most intense absorption peak, which was 665 nm for MB and 530 nm for EBT.

For single-solute rejection experiments, the drop in DI water flux after 50% recovery of feed water by the membrane was determined for feed water containing EBT dye, sodium chloride, and sodium sulfate. The following equation was used to determine the drop in DI water flux:

$$Drop\;in\;DI\;water\;flux=\frac{DI_{I}-DI_{F}}{DI_{I}}\times 100\%$$

where $DI_{I}$ is the initial DI water flux after precompaction and $DI_{F}$ is the DI water flux through the membrane after 50% recovery of feed water containing dye or salt.

#### 2.4.2. Multisolute Rejection Experiments

Influence of salinity on the dye and salt rejection performance of the membrane were observed using solutions prepared with 0.1 g/L EBT dye and varying concentrations (1, 5, 10, 25, 50 g/L) of sodium sulfate in the feed solution. Permeability and solute rejection were measured as mentioned before.

## 3. Results and Discussion

### 3.1. Membrane Characterizations

#### 3.1.1. Graphene Oxide Characterizations

**Figure 3:**
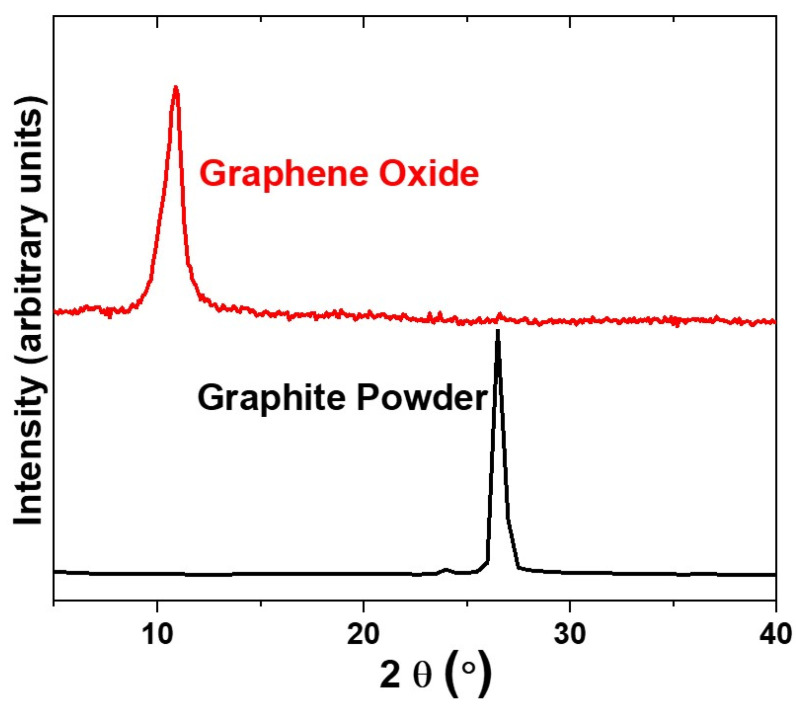
XRD pattern of the graphite powder starting reactant (**bottom**) and graphene oxide final product (**top**).

**Figure 4:**
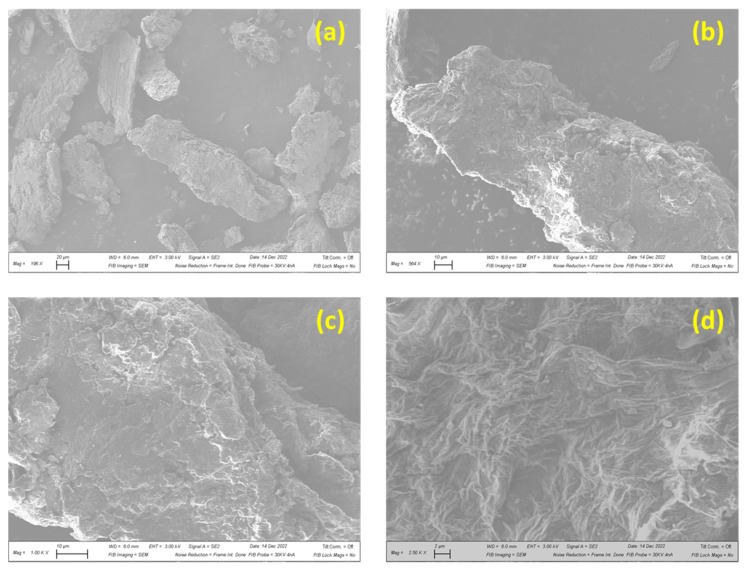
FESEM image of the GO obtained after synthesis at different magnifications: (**a**) 196×, (**b**) 564×, (**c**) 1000×, (**d**) 2500×.

In order to verify if the GO was successfully prepared from graphite powder, as described in the Section 2.1, XRD was used both in the graphite powder starting materials, and in the GO final product. Figure 3 shows the XRD pattern of the graphite powder on bottom and GO on top. It is noticeable that these XRD patterns are very different from each other, which confirms that the graphite powder was converted to GO. Additionally, the GO XRD pattern matches some references found in literature, where the peak, related to the (002) planes of the GO, is centered in 2-theta angle around 11°. This 2-theta angle corresponds to an interplanar spacing around 0.80 nm [30,31]. Figure 4 shows the microstructure of the GO particle using FESEM. At high magnification (2500×), graphene oxide demonstrates porous structure that was shaped by wrinkled thin-sheet formation (Figure 4d).

#### 3.1.2. Zeta Potential Measurement

**Figure 5:**
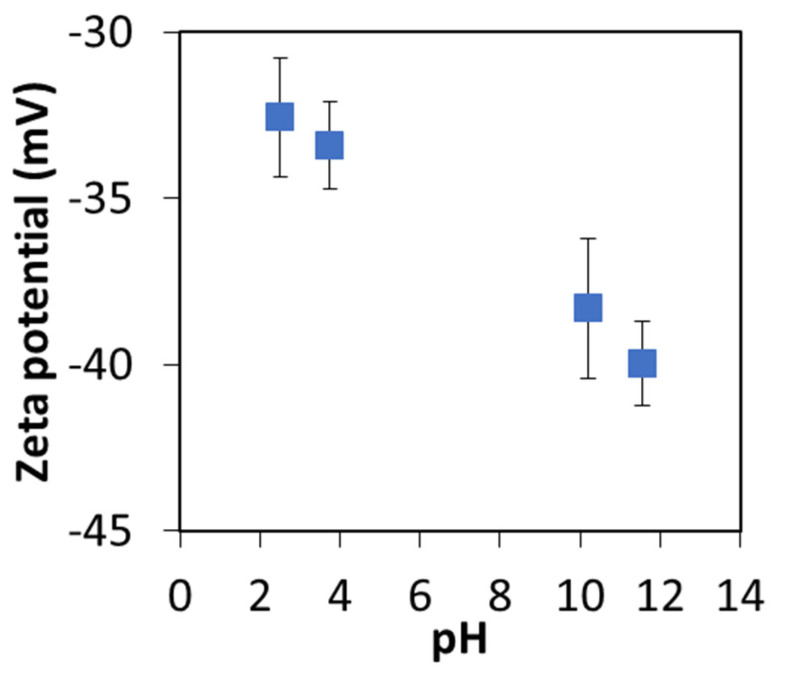
The zeta potential values of GO powders in aqueous media at different pH values.

The zeta potential of the GO powder was measured in aqueous suspension through a varied range of pH (Figure 5). Zeta potential provides a measure of the surface charge on a particle which influences the interaction with other particles in solution. Figure 5 indicates that the zeta potential on GO particles remains negative between pH 2.5 and 11.5. Hence, the GO particles will have an affinity toward binding to the positively charged amine functional groups present on PEI adsorbed layers.

#### 3.1.3. Surface Morphology and Hydrophilicity of GO–PEI-Modified CA Membrane

**Figure 6:**
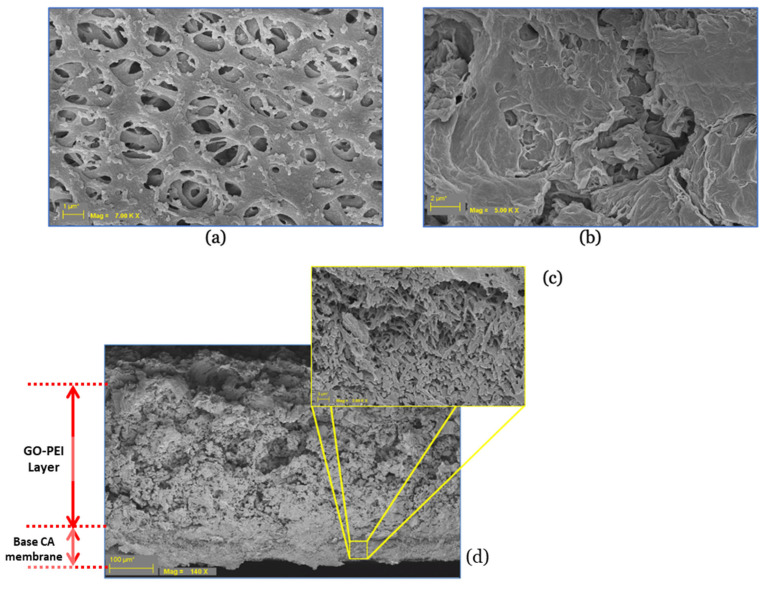
Surface morphology of a (**a**) pristine cellulose acetate membrane and (**b**) GO–PEI-modified cellulose acetate membrane, and cross-sectional images of the (**c**) pristine cellulose acetate membrane and (**d**) GO–PEI-modified cellulose acetate membrane.

The surface and cross-sectional morphologies of GO–PEI-modified CA membranes are shown in Figure 6.

The pristine CA base membrane demonstrated a typical porous surface (Figure 6a). The pressure-deposited GO–PEI covered the porous surface of the CA base membrane (Figure 6b). The cross-section profile indicates formation of a new skin layer on the base membrane (Figure 6c,d). It can be assumed that the electrostatic interaction between positively charged PEI and negatively charged GO together with the pressure deposition from vacuum filtration helped in forming a GO–PEI skin layer. The PEI molecules are not visible because branched PEI is a fully amorphous polymer that exists as a liquid at room temperature. As is evident from the cross-section in Figure 6d, we can see the interlinked GO–PEI layer, where the GO particles are connected with PEI molecules, on the surface of the base CA membrane. The thickness of the coating layer increased with the number of bilayers. Average thicknesses of the 1-bilayer, 2-bilayer, and 4-bilayer membranes were 0.32 ± 0.02, 0.66 ± 0.03, and 0.91 ± 0.03 mm, respectively. The average thickness of the base CA membrane was 0.14 ± 0.01 mm. For each type of membrane, a total of seven thickness readings were taken to obtain average and standard deviation values. The thickness of each by layer varied from 0.17 to 0.25 mm. This variation in thickness resulted due to the pressure applied on the membranes during thickness measurement. The static water contact angle of the base CA membrane was 43.25° ± 5.25° (total four measurements), and the water droplet rapidly dissipated in the membrane matrix. The measured water contact angle value is less than 90°, hence it indicates the CA membrane surface as hydrophilic [32]. The deposited GO–PEI bilayers form a dense porous surface on the base CA membrane. The water dispensed on the modified membranes immediately wicks into the porous GO–PEI bilayer(s) without forming any water droplet on the membrane surface. This indicates that the modified membrane surfaces were hydrophilic as the surfaces did not allow formation of any water droplets on the membrane surface due to immediate adsorption of water on the deposited layer.

The static water contact angle on a 2-bilayer-modified membrane after filtering 500 mL composite feed water (0.1 g/L EBT dye and 1 g/L sodium sulfate salt) was also observed. The membrane contact angle remained zero as the water droplet immediately wicked into the porous deposited bilayers. This observation indicates that the membrane surface remained hydrophilic after filtration. However, long-term operation of the membrane could influence the hydrophilicity of the membrane surface, which was beyond the scope of the present study. A relative comparison of hydrophilicity was not possible because the static water contact angle of the surface-modified membrane was zero both before and after the filtration experiment.

#### 3.1.4. Fourier Transform Infrared (FTIR) Spectroscopy Analysis

**Figure 7:**
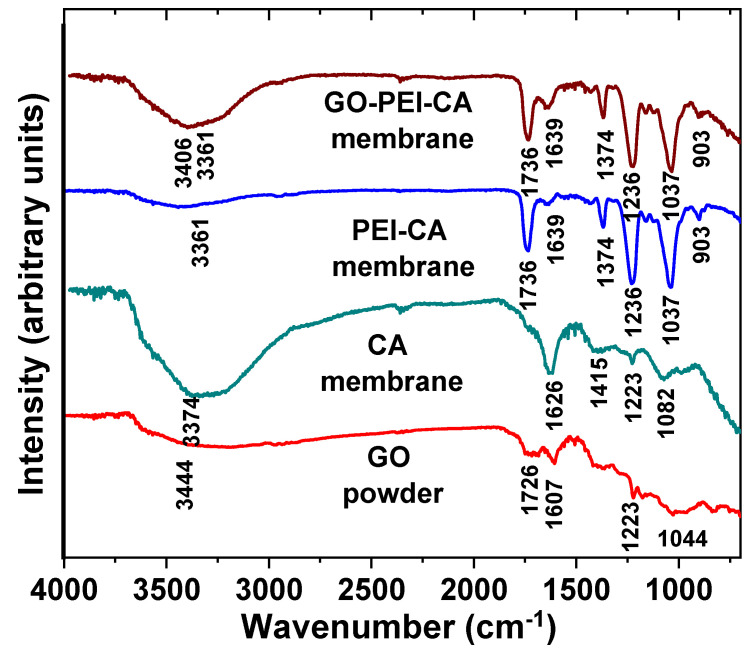
FTIR spectra of the graphene oxide powder, pristine cellulose acetate membrane, polyethyleneimine-soaked cellulose acetate membrane, and 2-bilayer GO–PEI-modified cellulose acetate membrane.

FTIR spectra of the GO, pristine CA membrane, PEI-soaked CA membrane, and final GO–PEI-modified membrane (2-bilayer) are given in Figure 7. The GO IR spectra presents bands at: 1044 cm^−1^, which is related to the deformation mode of C-O; 1223 cm^−1^, which is related to the stretching vibrations of C-OH; 1607 cm^−1^, which is related to the stretching vibrations of sp^2^ hybridized C=C; 1726 cm^−1^, which is related to the stretching vibrations of C=O; and 3444 cm^−1^, which is related to the stretching vibrations of O-H [33].

The cellulose acetate membrane presents the peaks at: 1082 cm^−1^, which is related to the deformation mode of C-O; 1223 cm^−1^, which is related to the stretching vibration of C-O; 1415 cm^−1^, which is related to the deformation mode of CH_2_; 1626 cm^−1^, which is related to the asymmetric stretching of C=O; and 3374 cm^−1^, which is related to the stretching of O-H [34].

When PEI is deposited onto the CA membrane, the bands related to the PEI become the most prevalent ones. For instance, the band at 1037 cm^−1^ is related to the C-N stretching vibrations and the band at 3361 cm^−1^ is related to the asymmetric stretching of the NH_2_ group [35]. This confirms the presence of positively charged NH_2_ functional groups on the membrane surface. Upon graphene oxide deposition onto the PEI/CA membrane, no substantial change is observed in comparison to the spectrum of the PEI/CA membrane. The most prominent change is based on the appearance of a broad band around 3406 cm^−1^, which can indicate the presence of the OH groups from the graphene oxide.

### 3.2. Selectivity of GO–PEI-Modified CA Membranes

**Figure 8:**
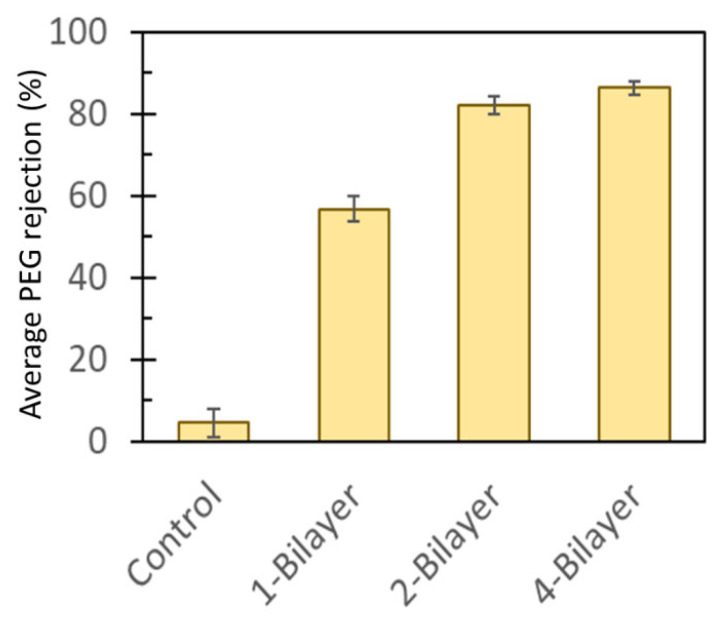
Comparison of the selectivity of 20 KD polyethylene glycol (PEG) of pristine CA membrane (control, 0.45 µm pore size) with that of GO–PEI surface-modified CA membranes.

Low molecular weight (20 KD) polyethylene glycol (PEG) solution was filtered through the membranes to assess membrane selectivity in a procedure for molecular weight cutoff (MWCO) analysis [29]. The average PEG rejections (%) increased with increasing numbers of bilayers (Figure 8). The surface-modified membranes showed superior rejection of PEG compared to the base CA membrane (0.45 µm pore size). The enhancement could be due to filling of the membrane pore spaces by GO particles, which enhanced rejection of PEG molecules, and adsorption of PEG molecules on the GO–PEI bilayers. From Figure 8, it is evident that the enhanced rejection was not linear, as between 2-bilayer and 4-bilayer membranes PEG rejection enhanced by only about 4.27% (81.96% and 86.23% for 2-bilayer and 4-bilayer surface-modified membranes, respectively). Thus, size exclusion of PEG molecules in smaller pore sizes can be assumed to play a more important role than adsorption in the rejection mechanism. Nonetheless, incorporation of GO–PEI bilayers exhibited substantial enhancement in PEG selectivity of the modified membranes.

### 3.3. DI Water Flux in Pristine CA and GO–PEI-Modified CA Membranes

**Figure 9:**
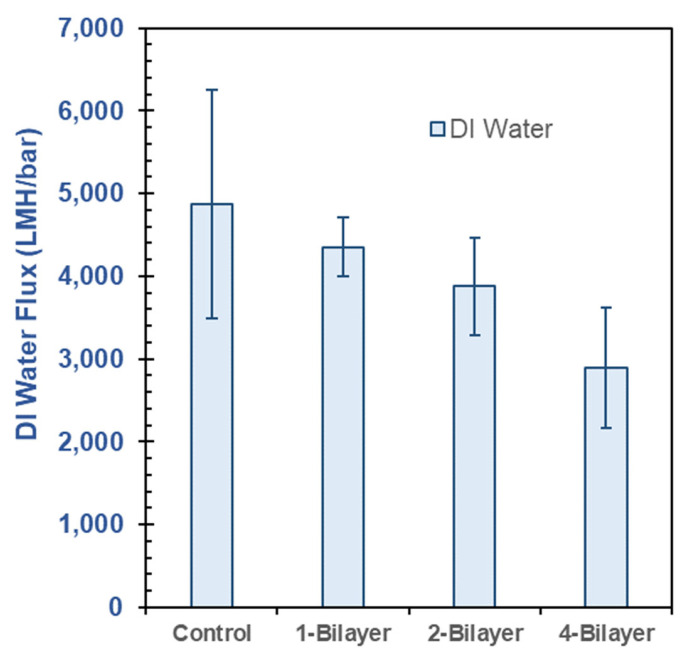
DI water flux performance of GO–PEI-modified CA membranes with varying bilayers.

The DI water flux of the membranes was measured in a cross-flow membrane filtration unit after the 60 min precompaction period, as shown in Figure 9. With increasing number of bilayers of GO–PEI deposition on the membrane surface the DI water flux was reduced. Increasing numbers of bilayers increased the membrane thickness and provided more resistance to flow through the membrane. Each bilayer deposition decreased the DI water flux by about 10%.

### 3.4. Individual Dye-Rejection Performance of the Membranes

**Figure 10:**
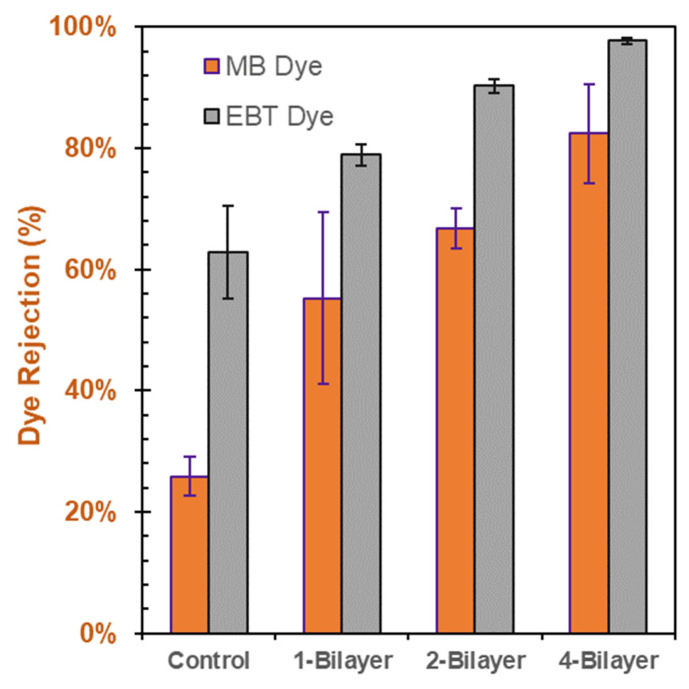
Individual dye-rejection performance of pristine CA membranes and GO–PEI-modified membranes with varying bilayers.

Methylene blue (MB) and eriochrome black T (EBT) dye solutions were employed at 0.1 g/L concentrations to form separate feed solutions in measuring the dye-rejection performance of the modified membranes with pristine CA membrane (control) and 1-, 2-, and 4-bilayer GO–PEI-modified membranes. The dye rejection performance of the modified membranes for both the MB and EBT solutions increased with an increasing number of bilayers. This relationship is illustrated in Figure 10. The enhanced dye rejection could be due to increased thickness of the membrane. The EBT dye demonstrated better rejection than the MB dye. This might be due the charges associated with the dyes and the charge on GO. The EBT dye is an anionic dye, while the MB dye is a cationic dye. GO has been reported to be negatively charged in aqueous solution [36]. The higher rejection performance of EBT could be linked to electrostatic repulsion between the negatively charged GO surface and anionic dye EBT. The cationic MB dye was attracted toward the negatively charged GO layer, and thus higher permeation was observed for MB dyes compared to EBT dyes at similar concentration. In the case of 2-bilayer GO–PEI-modified CA membranes, the membranes were able to achieve over 90% rejection of the EBT dye solution, compared to about 66% rejection of the MB dye.

### 3.5. Influence of Feed-Water Salinity or Dye Concentration on Membrane Performance

**Figure 11:**
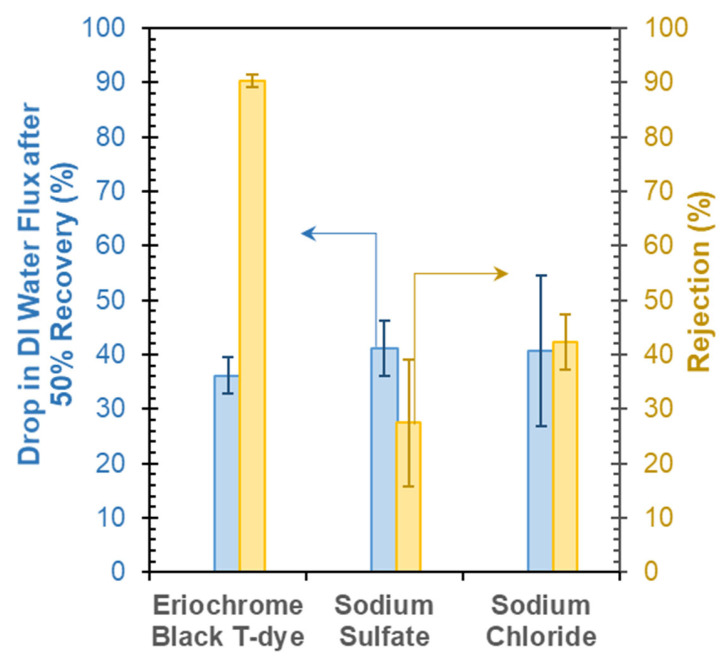
Comparison of dye- and salt-rejection performance and influence of 50% water recovery on DI water flux of 2-bilayer GO–PEI-modified CA membrane.

The effect of water recovery on membrane performance was evaluated by recovering 50% of the feed water at a given salt or dye content. Figure 11 indicates the percent reduction in DI water flux in the membrane from the same in a pristine membrane after recovering 50% feed water containing either 1 g/L salt (NaCl or Na_2_SO_4_) or 0.1 g/L EBT dye. The salt or dye rejection performance is also indicated in the figure. Between the two salts (i.e., NaCl and Na_2_SO_4_), Na_2_SO_4_ rejection (~27%) was smaller compared to NaCl (~42%). A similar drop in DI water fluxes (40–41%) was observed after 50% water recovery by the membrane for the salts. Such low rejections of inorganic salts were expected given the microfiltration membranes’ large pore size (0.45 µm). The less rejection of Na_2_SO_4_ salt compared to NaCl can be attributed to the relatively less electronegativity of sulfate (SO_4_^2−^) ion than chloride (Cl^−^) ion. As the GO deposited on the CA membrane has a negative surface charge, the chloride ions are rejected more than the sulfate ions due to electrostatic repulsion between two negatively charged entities.

Similarly, the anionic EBT dye also demonstrated higher rejection, as discussed previously. Compared to the salt ions, the larger size of the dye particles resulted in a more significant rejection (~90%) of dyes by the membrane. This also caused a larger drop in DI water flux, by around 36%. Deposition of dyes on the membrane induced surface and pore blocks of the membrane and reduced the DI water flux compared to the pristine membranes.

### 3.6. Influence on Feed-Water Salinity on Dye- and Salt-Rejection Performance

**Figure 12:**
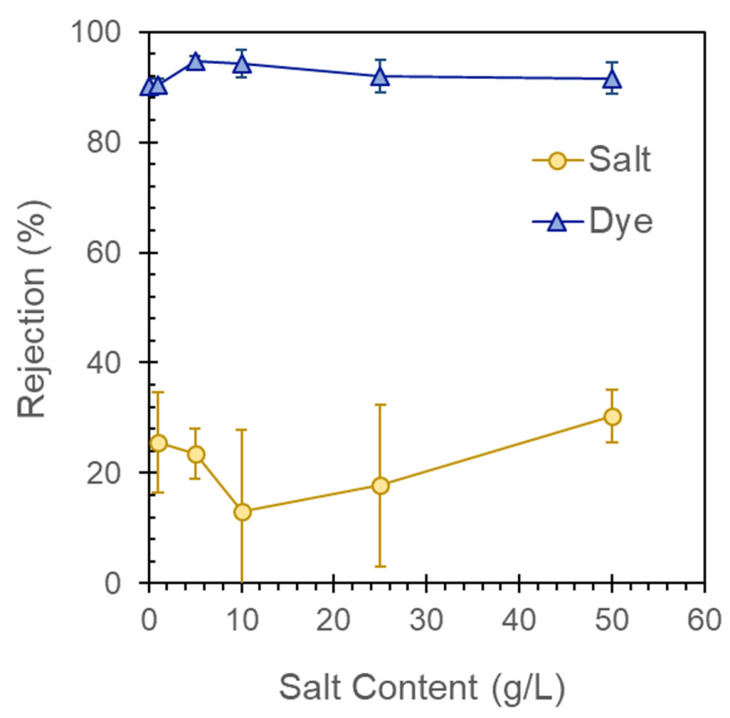
Impact of feed-water salinity on dye- and salt-rejection performance of 2-bilayer GO–PEI-modified CA membrane.

Figure 12 shows the salt and dye rejection performance of a two-bilayer GO–PEI-modified CA membrane with varying salinity and fixed dye concentration in a composite feed water. The EBT rejection was enhanced slightly in the composite feed-water experiments, possibly due to precipitation of salts on the membrane feed side or pore spaces and reducing the membrane pore sizes. The salt rejection performance varied between 13.0% and 30.3% in the composite feed-water experiments. The salt rejection performance declined with increasing feed salinity up to 10 g/L, possibly be due to salt concentration polarization on the membrane feed side. However, for higher feed salinity (25 and 50 g/L), the salt rejection performance improved, which may be due to salt precipitation blocking the membrane surface and pores.

### 3.7. Membrane Stability under Physical Stress

Membrane stability is an important issue in terms of application over a long-term period. In this study, a quick physical stress stability test was carried out to evaluate the stability of the GO–PEI bilayers on the membrane surface. The static water contact angle was zero after the physical stress test, indicating that the hydrophilicity was not hampered during the physical stress. Additionally, no visual deterioration of the deposits was observed after the physical stress test. We did not carry out chemical stress tests (by exposing the modified membranes in extreme acidic or basic solutions) because of potential degradation concerns of the base CA membrane. Based on the results in this study, the GO–PEI bilayer deposits were able to enhance dye rejection performance of the 0.45 µm CA membranes. Similar modifications can be made to other chemically resistant base membranes using GO–PEI for application in acidic or basic conditions.

It is very important to highlight the stability of membranes in long-term filtration experiments. While the membranes in the current study were subjected to only six to seven hours of operation (including precompaction) in a cross-flow module, long-term filtration experiments should be carried out to establish the stability of the deposits on the surface-modified membranes. The present study demonstrated enhanced rejection performance of the GO–PEI bilayer-modified membranes compared to the base CA membrane. However, the stability of the deposits over long-term filtration experiments was not assessed in this study. Potential wearing off of the deposits in long-term filtration experiments could necessitate a different deposition mechanism for the GO–PEI bilayers on the base membrane.

## 4. Conclusions

This work demonstrates the positive impact on dye rejection following surface modification of CA microfiltration membranes with GO–PEI bilayers. The layer-by-layer deposition of the positively charged PEI and negatively charged GO on the surface of a CA membrane facilitated the production of a thin-skin layer of GO–PEI on top of the base (CA) membrane. As increasing amounts of GO–PEI bilayers were deposited on the membrane, there was an observed increase in the resistance of flow through the membrane; a negative correlation between the number of GO–PEI bilayers deposited on the membrane and DI water flux was observed. Conversely, there was a positive correlation between the number of GO–PEI bilayers on the membranes and the observed dye-rejection performance; the dye-rejection performance of the modified membranes increased as 1-, 2-, and 4-bilayers of GO–PEI were deposited on the membranes. In particular, the anionic EBT dye saw superior rejection (~90% rejection) compared to the cationic MB dye (~80% rejection), presumably due to the electrostatic repulsion between the negatively charged GO surface and anionic EBT dye. On the other hand, modified membranes seemed to better reject NaCl (~42%) than Na_2_SO_4_ (~27% rejection), which may be due to the relatively less electronegative nature of the SO_4_^2−^ ion compared to that of the Cl^−^ ion.

The modified membranes were further studied with respect to their response to varying feed-water salinity and/or dye concentrations. The effect of water recovery on the membrane performance was evaluated after a 50% recovery of the feed water at predetermined salt and dye concentrations: after 50% recovery of the saline and dye-laden feed water, there was an observed drop in DI water fluxes of ~40–41% and 36%, respectively. The studies also showed a slight increase in EBT dye-rejection during the composite feed-water experiments, possibly due to the precipitation of salts on the membrane feed side or pore spaces, subsequently reducing the membrane pore sizes. Meanwhile, the salt rejection performance varied between 13.0 and 30.3% and declined with increasing feed salinity of up to 10 g/L, which may be due to the concentration polarization on the membrane feed side, and then increased for the higher feed salinities of 25 and 50 g/L, which may be due to the salt precipitation mentioned previously. Ultimately, the results of this study point toward the need to investigate the treatment of more complex feed water with varying salinity, but, overall, the results of this work hold great promise in terms of the increased rejection due to the surface modification of the GO–PEI membranes.

## Data Availability

The data presented in this study are available on request from the corresponding author.
